# Oncological Outcomes and Safety Profiles of Adjuvant Nivolumab in Muscle‐Invasive Urothelial Carcinoma

**DOI:** 10.1111/iju.70664

**Published:** 2026-10-19

**Authors:** Satoshi Katayama, Tatsushi Kawada, Keiichiro Mori, Kosuke Iwatani, Shota Inoue, Tomofumi Watanabe, Ichiro Tsuboi, Tomoaki Yamanoi, Takehiro Iwata, Kazuma Tsuboi, Wataru Murao, Tetsuya Nakada, Kohei Ogawa, Akihiro Matsukawa, Takafumi Yanagisawa, Shingo Nishimura, Kensuke Bekku, Koichiro Wada, Takahiro Kimura, Motoo Araki

**Affiliations:** ^1^ Department of Urology Okayama University Graduate School of Medicine, Dentistry and Pharmaceutical Sciences Okayama Japan; ^2^ Department of Urology The Jikei University School of Medicine Tokyo Japan; ^3^ Department of Urology Hiroshima City Hiroshima Citizens Hospital Hiroshima Japan; ^4^ Department of Urology National Hospital Organization Iwakuni Clinical Center Yamaguchi Japan; ^5^ Department of Urology Shimane University Hospital Shimane Japan

**Keywords:** adjuvant nivolumab, eosinophil, hypophysitis, upper tract urothelial carcinoma, urothelial carcinoma

## Abstract

**Objectives:**

Perioperative immunotherapy has become the standard of care in patients with high‐risk muscle‐invasive urothelial carcinoma (MIUC). This study aimed to clarify oncological outcomes and safety profiles of adjuvant nivolumab therapy in MIUC, focusing on upper tract urothelial carcinoma (UTUC).

**Methods:**

This multicenter study comprised 97 patients undergoing removal of primary tumors, followed by adjuvant nivolumab therapy with a potential follow‐up period ≥ 12 months. Patients were divided according to the primary tumor location: bladder cancer (BC), *n* = 66 (68%); UTUC, *n* = 31 (32%). Multivariable Cox regression analyses were applied to identify prognostic factors for disease‐free survival (DFS).

**Results:**

Before surgery, 38% of patients had an estimated glomerular filtration rate (eGFR) < 50 mL/min/1.73m^2^, but this proportion increased to 57% at nivolumab initiation with 19% of UTUC having eGFR < 30. The overall DFS at 6 months was 61.5%. DFS did not differ significantly between BC and UTUC (*p* = 0.83). On multivariable analyses, a higher absolute eosinophil count was significantly associated with worse DFS (HR 2.24; 95% CI, 1.24–4.03, *p* = 0.007), while primary tumor location was not. Immune‐related adverse events (irAEs) of adrenal insufficiency and hypophysitis occurred in 8% and 4% of patients, respectively, with grade ≥ 3 occurring at 3.3–18.3 months.

**Conclusions:**

Adjuvant nivolumab therapy may provide comparable oncological outcomes between BC and UTUC. Baseline eosinophil count was an independent prognosticator for DFS. The incidences of adrenal insufficiency and hypophysitis appeared higher in our real‐world data. Clinicians should recognize these irAEs due to nonspecific symptoms and considerable variability in time of onset.

## 1 Introduction

Radical surgery with/without neoadjuvant chemotherapy (NAC) is the mainstay of treatment for patients with muscle‐invasive urothelial carcinoma (MIUC), which encompasses bladder cancer (BC) and upper tract urothelial carcinoma (UTUC). However, pathologically confirmed extravesical disease or lymph node metastasis have dismal survival outcomes, with 5‐year recurrence‐free survival (RFS) and overall survival (OS) rates of 30%–52% and 26%–45%, respectively, even in the contemporary era [1]. With the goal of improving survival outcomes of patients at higher risk of recurrence, CheckMate 274, a Phase III randomized controlled trial (RCT) evaluating adjuvant nivolumab for patients with high‐risk UC after extirpative surgery, showed that the adjuvant nivolumab conferred a significant disease‐free survival (DFS) benefit versus placebo (20.8 vs. 10.8 months, respectively) [2]. In the 3‐year extended follow‐up results, a continuous DFS benefit was observed, with an interim OS analysis favoring nivolumab over placebo in the intention‐to‐treat (hazard ratio [HR], 0.76; 95% confidence interval [CI], 0.61–0.96) [3]. However, given a limited proportion of UTUC (21%) enrolled in CheckMate 274, some have questioned the efficacy of adjuvant nivolumab for UTUC regardless of NAC exposure. Moreover, in addition to several promising biomarkers (e.g., combined positive score [CPS] and circulating tumor DNA [ctDNA]) [4, 5], absolute eosinophil count (AEC) has been reported to have prognostic potential in other malignancies [6, 7]. However, the role of AEC remains uninvestigated in MIUC treated with adjuvant nivolumab therapy. To clarify these uncertainties, this multicenter study analyzed the oncological outcomes and safety profiles of adjuvant nivolumab following extirpative surgery in patients with high‐risk MIUC, with particular emphasis on UTUC for a better understanding of real‐world practice.

## 2 Materials and Methods

### 2.1 Patient Selection and Study Protocol

This retrospective multicenter study included 126 patients with high‐risk MIUC treated with radical cystectomy (RC) and/or radical nephroureterectomy (RNU), followed by adjuvant nivolumab therapy between May 2022 and May 2025 at 15 hospitals, including Okayama University Hospital, the Jikei University Hospital, and their affiliated hospitals. All patients had histologically confirmed pure or predominant urothelial carcinoma of the bladder or upper urinary tract. Eligible patients had pT3‐pT4a and/or pN+, or ypT2‐ypT4 and/or ypN+ disease if they received NAC according to CheckMate 274. No patient had metastatic disease at the time of nivolumab initiation. The exclusion criteria were as follows: patients with distant lymph node metastasis at the time of extirpative surgery, those whose tumors were of a pure or predominant histological subtype, and those who received any prior therapy not covered by the insurance. Additionally, patients without recurrence or death who had less than 1 year of potential follow‐up were also excluded because adjuvant nivolumab was scheduled to be administered for 1 year. Adjuvant nivolumab therapy was administered intravenously (240 mg every 2 weeks or 480 mg every 4 weeks). Treatment was continued until either completion of 12 months, development of recurrence, or occurrence of immune‐related adverse events (irAEs). Patients were divided into BC and UTUC (including ureteral and/or pelvic cancer) groups. If patients had both BC and UTUC, the more advanced tumor location eligible for receipt of adjuvant nivolumab therapy was selected as the primary site. This study was approved by the ethics committee at all participating institutions (approval number: K2505‐009 in Okayama University Hospital).

### 2.2 Surgical Management and Follow‐Up

Patients with MIBC and UTUC underwent RC and RNU with excision of the bladder cuff, with or without standard lymphadenectomy, respectively. The choice of surgical approach (open, laparoscopic or robotic), urinary diversion (orthotopic ileal neobladder, ileal conduit, or cutaneous ureterostomy), and extent of lymphadenectomy were decided based on patients' condition and/or institutional treatment strategy.

### 2.3 Outcome Assessment

The primary outcome of interest was DFS. The secondary outcomes of interest included safety profiles, non‐urothelial tract recurrence‐free survival (NUTRFS), and OS. DFS, NUTRFS, and OS were defined as the time from the date of adjuvant nivolumab initiation to the date of first recurrence (including intravesical, local, or distant) or death, to the date of first nonurothelial recurrence (including local nonurothelial tract or distant) or death, or to the date of death from any cause, respectively. As data were collected in a retrospective manner, a single common data cutoff date was not applied; patients without an event were censored at the date of last follow‐up at each participating institution. The last follow‐up dates ranged from March 2025 to April 2026 across institutions. AEC was assessed at the time of adjuvant nivolumab initiation. The severity of AEs was graded according to the Common Terminology Criteria for Adverse Events version 5.0.

### 2.4 Statistical Analysis

Categorical variables are presented as frequencies and percentages, while continuous variables as medians and interquartile ranges (IQRs). Differences between categorical and continuous variables were assessed using the chi‐square and the Mann–Whitney *U* tests, respectively. The Kaplan–Meier method was applied to graphically visualize DFS, NUTRFS, and OS between BC and UTUC. Cox regression analyses were applied to determine prognostic factors for patients who received adjuvant nivolumab after surgery, with widely recognized confounding factors adjusted for in the multivariable analysis. The optimal cutoff of AEC was determined by creating a time‐dependent receiver operating characteristic curve to yield the highest Youden index value at 12 months (< 239.7 vs. ≥ 239.7/μL). To minimize the potential effects of shorter follow‐up duration, we performed sensitivity analysis restricted to patients who initiated adjuvant nivolumab at least 1 year before the data cut‐off date at each institution. In addition, we performed a restricted cubic spline to examine and visualize possible nonlinear association of AEC as a continuous variable with DFS. Statistical significance was set at *p* < 0.05. All tests were two‐sided. Analyses were performed using R version 3.6.3 (R Foundation for Statistical Computing, Vienna, Austria) and Stata/MP 14.2 statistical software (Stata Corp., College Station TX, USA).

## 3 Results

### 3.1 Patient Characteristics

After applying the exclusion criteria (Supplementary Figure 1), 97 patients met the inclusion criteria for this study; 31 (32%) were located in the upper tract, and 66 (68%) in the bladder. The distribution of patients' characteristics is shown in Table 1. At the initial visit, 38% of cases had an estimated glomerular filtration rate (eGFR) < 50 mL/min/1.73 m^2^, but a 19% increase in the proportion of those with eGFR < 50 (57%) was observed at the initiation of nivolumab therapy; especially, 19% of UTUC was deemed ineligible to platinum‐based chemotherapy (eGFR < 30).

**TABLE 1:** Patients' characteristics according to the tumor location (bladder cancer vs. upper tract urothelial carcinoma).

	Total, *N* = 97	Bladder cancer, *N* = 66 (68%)	Upper tract urothelial carcinoma, *N* = 31 (32%)	*p*
Sex, no. (%)				0.74
Male	77 (79)	53 (80)	24 (77)	
Female	20 (21)	13 (20)	7 (23)	
Age, median (IQR)	72 (66–75)	72 (66–75)	70 (64–75)	0.63
ECOG‐PS, no (%)				0.44
0	85 (88)	59 (89)	26 (84)	
1	12 (12)	7 (11)	5 (16)	
eGFR (mL/min/1.73 m^2^) at the initial visit, no. (%)				0.69
< 30	4 (4)	2 (3)	2 (6)	
30–49.9	33 (34)	22 (33)	11 (36)	
≥ 50	60 (62)	42 (64)	18 (58)	
eGFR (mL/min/1.73 m^2^) at nivolumab initiation, no. (%)				**< 0.001**
< 30	10 (10)	4 (6)	6 (19)	
30–49.9	45 (47)	24 (36)	21 (68)	
≥ 50	42 (43)	38 (58)	4 (13)	
Absolute eosinophil count (/μL), no. (%)				0.45
< 239.7	56 (58)	40 (61)	16 (52)	
≥ 239.7	36 (37)	23 (35)	13 (42)	
Unknown	5 (5)	3 (5)	2 (6)	
Surgical approach, no. (%)				**< 0.001**
Robotic	65 (67)	56 (85)	9 (29)	
Laparoscopic	29 (30)	8 (12)	21 (68)	
Open	3 (3)	2 (3)	1 (3)	
Pathological tumor stage				0.09
≤ pT1	5 (5)	5 (8)	0 (0)	
pT2	9 (9)	8 (12)	1 (3)	
≥ pT3	83 (86)	53 (80)	30 (97)	
Pathological nodal stage				**< 0.001**
pN0	27 (27)	22 (33)	5 (16)	
pN1	19 (20)	16 (24)	3 (10)	
≥ pN2	19 (20)	19 (29)	0 (0)	
pNx	32 (33)	9 (14)	23 (74)	
Histological subtype, no. (%)				0.95
No	63 (65)	43 (65)	20 (65)	
Yes	34 (35)	23 (35)	11 (35)	
Squamous differentiation	18 (19)	12 (18)	6 (19)	
Sarcomatoid differentiation	5 (5)	2 (3)	3 (10)	
Glandular differentiation	4 (4)	2 (3)	2 (6)	
Plasmacytoid differentiation	3 (3)	3 (5)	0 (0)	
Micropapillary differentiation	2 (2)	2 (3)	0 (0)	
Others	4 (4)	2 (3)	2 (6)	
Neoadjuvant chemotherapy, no (%)				**< 0.001**
No	31 (32)	12 (18)	19 (61)	
Yes	66 (68)	54 (82)	12 (39)	
Gemcitabine + cisplatin	47 (48)	38 (60)	9 (29)	
Gemcitabine + carboplatin	11 (11)	10 (16)	1 (7)	
Dose‐dense MVAC	2 (2)	0 (0)	2 (4)	
Others	6 (6)	6 (8)	0 (0)	

*Note:* Bold *p* values are considered statistically significant.

Abbreviations: ECOG‐PS Eastern Cooperative Oncology Group‐performance status; MVAC, methotrexate, vinblastine, doxorubicin, and cisplatin.

### 3.2 Oncological Outcomes

During the median follow‐up of 17.0 (IQR: 12.7–24.6) months, 50 patients (52%) recurred; 42 (84%) during adjuvant nivolumab and 16% after nivolumab termination (Table 2). The top sites that suffered recurrence were lymph node (38%), followed by peritoneum/carcinomatosis (34%) and lung (22%); two (4%) experienced intravesical recurrence. Thereafter, 41 (42%) patients proceeded with subsequent treatment (Supplementary Table 1). Kaplan–Meier analyses estimated that DFS estimate at 6 months was 61.5% (95% CI, 51.0–70.4) (Figure 1). With regard to the primary location, DFS (6 months: UTUC 64.5% vs. BC 60.1%, *p =* 0.83), NUTRFS (*p =* 0.43), and OS (*p =* 0.49) did not differ significantly between the BC and UTUC groups (Figure 2). On the multivariable analyses, elevated AEC (HR 2.24; 95% CI, 1.24–4.03; *p =* 0.007) as well as pN positivity (HR 2.26; 95% CI, 1.10–4.67; *p =* 0.027) remained associated with worse DFS, meanwhile primary location was not associated with DFS (HR 1.18; 95% CI 0.55–2.53) (Table 3). A linear relationship between AEC and DFS was observed (Figure 3). A significant association of AEC with DFS disappeared in a subgroup of patients excluding those without lymph node dissection (pNx) (Supplementary Table 2). In subgroup analyses, NAC exposure was not associated with DFS in the whole, BC and UTUC groups, respectively (Supplementary Figure 2). Moreover, patients harboring two risk factors (≥ ypT2/pT3 and (y)pN+) showed worse DFS than those having only the risk factor of T stage (≥ ypT2/pT3 and (y)pN0) (*p =* 0.029) (Supplementary Figure 3).

**TABLE 2:** Clinical outcomes of patients who received adjuvant nivolumab according to the tumor location (bladder vs. upper tract urothelial carcinoma).

	Total	Bladder cancer	Upper tract urothelial carcinoma	*p*
	*N* = 97	*N* = 66 (68%)	*N* = 31 (32%)	
Follow‐up duration (months), median (IQR)	17.0 (12.7–24.6)	17.5 (12.0–25.1)	16.8 (13.6–20.7)	0.64
Treatment status of nivolumab, no (%)				0.49
Ongoing	0 (0)	0 (0)	0 (0)	
Discontinuation	55 (57)	39 (59)	16 (52)	
Completion of scheduled therapy	42 (43)	27 (41)	15 (48)	
Reason for discontinuation, no (%)				0.53
Recurrence	34 (35)	23 (35)	11 (35)	
Adverse events	17 (18)	12 (18)	5 (16)	
Others	4 (4)	4 (6)	0 (0)	
Recurrence, no (%)				0.67
No	47 (48)	31 (47)	16 (52)	
Yes	50 (52)	35 (53)	15 (48)	
During nivolumab	42 (84)	27 (77)	15 (100)	
After finishing nivolumab	8 (16)	8 (23)	0 (0)	
Recurrence site, no (%)				
Lymph node	19 (38)	13 (37)	6 (40)	
Peritoneum/carcinomatosis	17 (34)	12 (34)	5 (33)	
Lung	11 (22)	6 (17)	5 (33)	
Bone	9 (18)	9 (26)	0 (0)	
Liver	7 (14)	7 (20)	0 (0)	
Intravesical	2 (4)	0 (0)	2 (13)	

**FIGURE 1:**
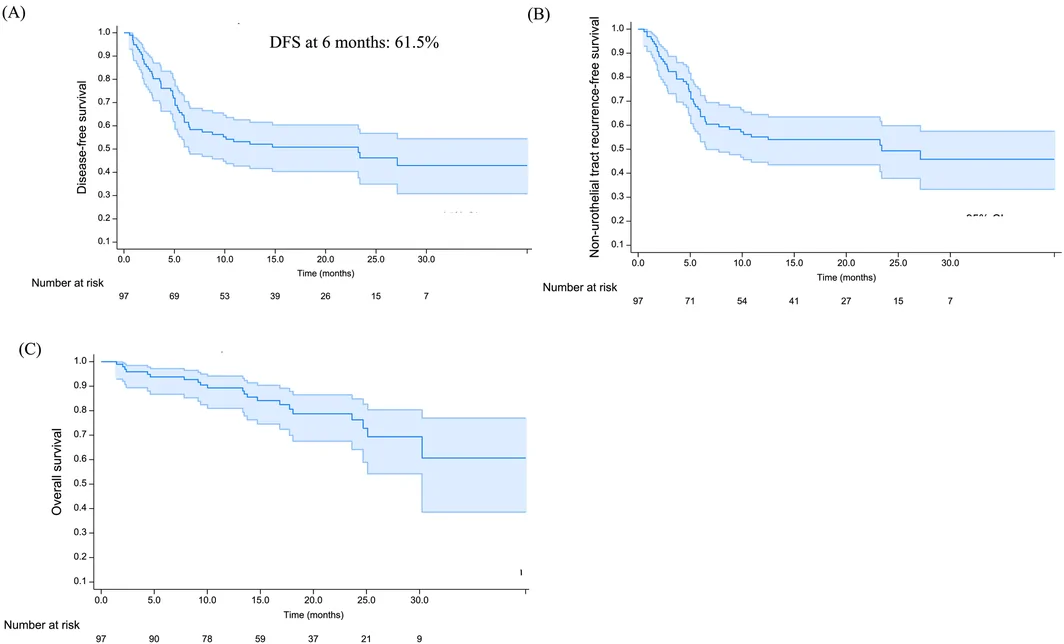
Kaplan–Meier analysis of disease‐free (A) and non‐urothelial tract recurrence‐free (B), and overall survival (C) in patients with high‐risk muscle‐invasive urothelial carcinoma treated with adjuvant nivolumab therapy following extirpative surgery.

**FIGURE 2:**
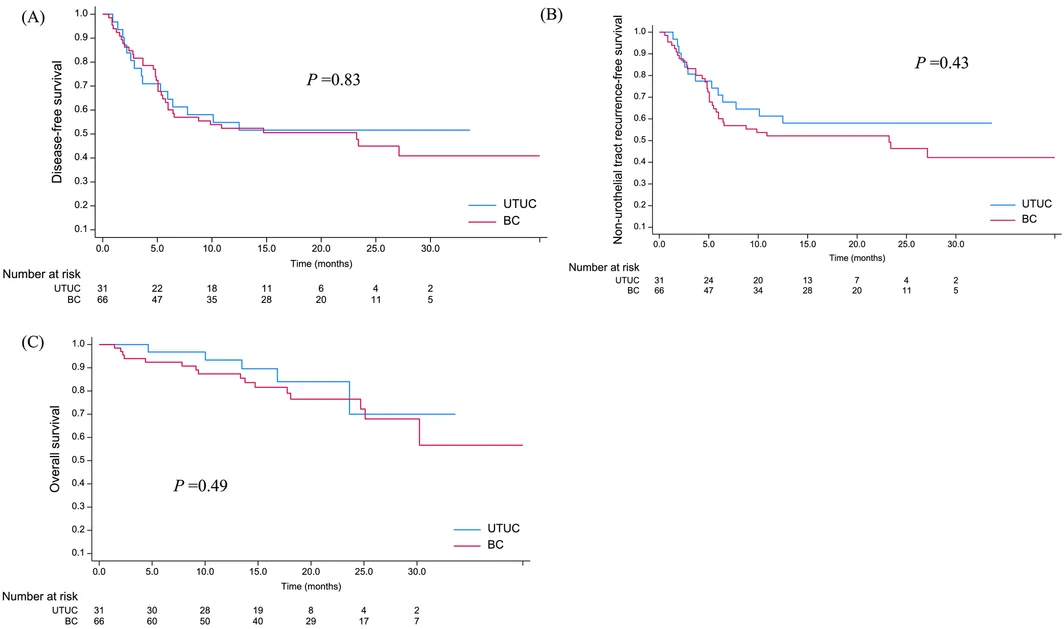
Kaplan–Meier analysis of disease‐free (A) and non‐urothelial tract recurrence‐free (B), and overall survival (C) according to tumor location (bladder cancer vs. upper tract urothelial carcinoma) in patients with high‐risk muscle‐invasive urothelial carcinoma treated with adjuvant nivolumab therapy following extirpative surgery.

**TABLE 3:** Univariable and multivariable analyses assessing factors associated with disease‐free survival in patients with high‐risk urothelial carcinoma treated with adjuvant nivolumab.

	DFS					
	Univariable			Multivariable		
	HR	95% CI	*p*	HR	95% CI	*p*
Age (ref. < 70)	0.71	0.41–1.23	0.22			
Sex	0.67	0.31–1.43	0.30			
ECOG‐PS 1 (ref. 0)	1.85	0.86–3.97	0.12			
Neoadjuvant chemotherapy	0.84	0.47–1.49	0.55	0.72	0.35–1.45	0.35
Pathological T stage (ref. ≤ ypT1/pT2)	1.15	0.36–3.70	0.82	1.23	0.34–4.43	0.75
Pathological N stage (ref. N0/Nx)	1.79	1.03–3.13	**0.039**	2.26	1.10–4.67	**0.027**
eGFR at nivolumab initiation (ref. < 50)	0.73	0.41–1.28	0.27			
Histological subtype	1.03	0.58–1.83	0.93			
Absolute eosinophil count (ref. < 239.7/μL)	2.25	1.26–4.00	**0.006**	2.24	1.24–4.03	**0.007**
UTUC (ref. bladder)	0.93	0.51–1.71	0.82	1.18	0.55–2.53	0.87

*Note:* Bold *p* values are considered statistically significant.

Abbreviations: DFS, disease‐free survival; eGFR, estimated glomerular filtration rate; UTUC, upper tract urothelial carcinoma.

**FIGURE 3:**
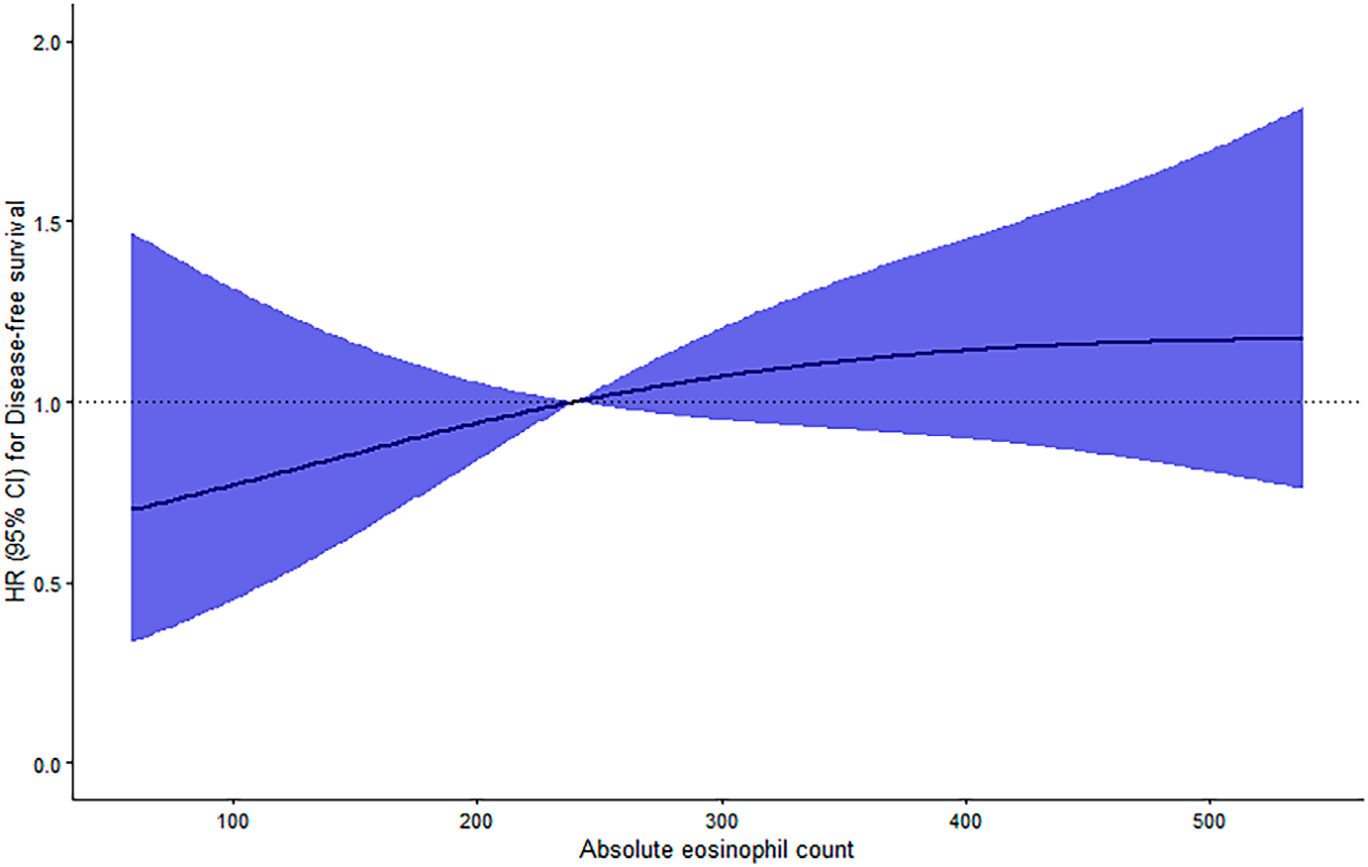
Association of absolute eosinophil count at the time of adjuvant nivolumab initiation with disease‐free survival, visualized by restricted cubic spline. An absolute eosinophil count of 239.7 was used as the reference value.

### 3.3 Safety Profiles

During the follow‐up period, 37 patients (38%) experienced irAEs of all grades, of whom 17 (18%) experienced irAEs of Grade 3 or higher. The most common irAEs of all grades related to nivolumab therapy were endocrine disorders (21%), including adrenal insufficiency (8%), hypophysitis (4%), and hypothyroidism (5%). Three cases were diagnosed with adrenocorticotropic hormone (ACTH) deficiency (3%), all of whom were Grade 3 or higher. Similarly, the most common irAEs of Grade 3 or higher were endocrine disorders (8%), which consisted of adrenal insufficiency (4%) and hypophysitis (4%) (Table 4). There was no death associated with irAEs from nivolumab therapy. The median time to Grade 3 or higher of adrenal insufficiency/hypophysitis from the initiation of adjuvant nivolumab was 5.9 months, ranging from 3.3 to 18.3 months. The frequency of irAEs of Grade 3 or higher was identical for patients with BC and UTUC (*p =* 0.41).

**TABLE 4:** Immune‐related adverse events of patients with high‐risk urothelial carcinoma who received adjuvant nivolumab.

			≥ Grade 3, no (%)		
	Any grade, no. (%)	≥ Grade 3, no. (%)	Bladder cancer	Upper tract urothelial carcinoma	*p*
	*N* = 97	*N* = 97	*N* = 66 (68%)	*N* = 31 (32%)	
Number of patients who experienced adverse events, no. (%)	37 (38)	17 (18)	13 (20)	4 (13)	0.41
Endocrine disorder	20 (21)	8 (8)	5 (8)	3 (10)	
Adrenal insufficiency	8 (8)	4 (4)	2 (3)	2 (6)	
Hypophysitis	4 (4)	4 (4)	3 (5)	1 (3)	
Hypothyroidism	5 (5)	0 (0)	0 (0)	0 (0)	
Thyroiditis	3 (3)	0 (0)	0 (0)	0 (0)	
Skin disorder	8 (8)	4 (4)	4 (6)	0 (0)	
Interstitial pneumonia	4 (4)	1 (1)	1 (2)	0 (0)	
Increased liver/biliary tract enzyme level	2 (2)	1 (1)	0 (0)	1 (3)	
Renal dysfunction	3 (3)	2 (2)	2 (3)	0 (0)	
Colitis	1 (1)	0 (0)	0 (0)	0 (0)	
Arthritis	2 (2)	1 (1)	1 (2)	0 (0)	
Others	3 (3)	0 (0)	0 (0)	0 (0)	

## 4 Discussion

In this multi‐institutional study, we assessed the real‐world utility and safety profiles of adjuvant nivolumab following extirpative surgery in patients with MIUC. Our findings revealed that adjuvant nivolumab therapy for UTUC conferred comparable survival outcomes regarding DFS, NUTRFS, and OS between UTUC and BC. Furthermore, AEC was an independent prognostic factor for DFS. Meanwhile, we clarified the real‐world safety profiles of adjuvant nivolumab therapy in this population, noting that irAEs of adrenal insufficiency and hypophysitis occurred more frequently than in CheckMate 274 (only secondary adrenocortical insufficiency was reported as 0.3%).

Nivolumab was approved by the US Food and Drug Administration in August 2021 for the adjuvant treatment of patients with UC who are at high risk of recurrence after extirpative surgery. Controversy remains regarding the role of adjuvant immune checkpoint inhibitors (ICIs) after radical surgery, given the conflicting results from three recent RCTs: CheckMate 274 (nivolumab), IMvigor010 (atezolizumab), and AMBASSADOR (pembrolizumab) [2, 8, 9]. However, recent meta‐analyses including these RCTs showed a 23%–24% reduction in the risk of recurrence or death, supporting the use of ICIs in the adjuvant setting [10, 11, 12]. Indeed, cumulative evidence suggests that patients with MIBC harboring significant residual disease (≥ ypT2 and/or ypN1‐3) at the time of RC after NAC had poor 5‐year cancer‐specific survival and OS (36%–60%, and 32%–59%, respectively) [13], highlighting the strong prognostic impact of significant residual chemoresistant tumors and the pragmatic need for other adjuvant therapies with different mechanisms of action for this population. We showed that adjuvant nivolumab therapy exhibited DFS at 6 months of 61.5% in the real‐world setting, which was inferior to CheckMate 274 findings (DFS at 6 months: 74.9%) [2].

Driven by the approval of ICI use in the perioperative setting as well as the metastatic setting for UC, efforts have been made to identify predictive biomarkers of ICIs. The post hoc analysis of CheckMate 274 conducted by Galsky et al. [4] explored the potential value of CPS (the measurement of PD‐L1 expression on both tumor cells [TC] and immune cells surrounding the tumor). They revealed that 81% of patients with PD‐L1 < 1% on TCs had CPS ≥ 1%, for whom the DFS benefit was nearly doubled in comparison to placebo (19 vs. 10 months). This finding might suggest that CPS measurement, rather than PD‐L1 alone, is more important for identifying optimal candidates who benefit from adjuvant nivolumab therapy. Additionally, ctDNA offers the promising discriminatory ability to guide individualized treatment strategies; patients without detectable ctDNA had > 10‐fold higher median DFS compared with those with detectable ctDNA in the exploratory analysis of CheckMate 274 [5]. Although these promising biomarkers have been investigated, readily available clinical biomarkers associated with the outcomes of adjuvant nivolumab remain uninvestigated. Thus, we examined prognostic factors for adjuvant nivolumab and found that AEC was inversely associated with DFS. Previous research explored the impact of eosinophil count on oncological outcomes, with varying results. Multiple studies, in accordance with our study, found that a high eosinophil count conferred an increased risk of mortality in non‐small cell lung cancer and head and neck squamous cell carcinoma receiving ICIs [14, 15]. In contrast, positive correlations between eosinophilia and survival outcomes were observed in metastatic urothelial carcinoma and other malignancies [16, 17]. Emerging evidence suggests that eosinophils can amplify tumor immunity through direct and indirect mechanisms. Their release of cytokines and granule contents, and the binding of eosinophil receptors to tumor microenvironment (TME) and TCs alter oncological outcomes [18]. On the other hand, eosinophils can modulate local recruitment of Treg cells, resulting in increased tumor colonization and promoting tumor growth [19]. Given the pleiotropic role of eosinophils in the TME, outcomes may vary widely depending on cancer type, measurement timing (prior to or during treatment), or treatment setting (adjuvant or metastatic), etc. In addition, the biological mechanisms underlying this association remain under research. Since ICI monotherapy or in combination with chemotherapies is central in the management of MIUC, further understanding of the causal relationship between eosinophilia and cancer immunotherapy will help refine individualized treatment strategies.

To date, the optimal adjuvant treatment strategy for UTUC remains a matter of debate. Despite the small number of patients in this study precluding robust evidence, adjuvant nivolumab for UTUC showed DFS comparable to that of BC. The POUT trial, which compared adjuvant cisplatin‐ or carboplatin‐based chemotherapy with surveillance in patients with high‐risk UTUC at RNU, demonstrated a meaningful DFS benefit (5‐year: 62% vs. 45%, respectively), providing Level 1 evidence according to the guidelines [20]. Even though the strong guideline recommendation has been advocated, postoperative complications, renal impairment, and/or comorbidities hinder the implementation of adjuvant chemotherapy into our daily practice. Indeed, the proportion of patients with UTUC ineligible for platinum‐based chemotherapy (eGFR < 30) at the time of diagnosis was 6%, which increased to 19% at the nivolumab initiation. Although caution should be exercised owing to selection bias (i.e., the exclusion of patients receiving adjuvant chemotherapy or surveillance), a considerable number of patients are assumed to be cisplatin‐ineligible after surgery. Given the substantial gap between the evidence and our real‐world practice, adjuvant nivolumab may be proposed as a practical option for patients with locally advanced UTUC ineligible for adjuvant chemotherapy, ideally for select patients with CPS ≥ 1% or possibly with detectable ctDNA [5, 21]. However, further well‐designed studies are necessary to ensure the validity of these treatment strategies.

Surprisingly, we found that incidences of endocrine disorders were 21%, with 8% of adrenal insufficiency and 4% of hypophysitis. Given that adrenal insufficiency generally occurs as a primary disorder or secondary to hypophysitis, and urooncologists may not have sufficient professional expertise to recognize endocrine irAEs, several primary cases in our study may have harbored secondary adrenal insufficiencies from hypophysitis. Previous data showed programmed cell death‐1 (PD‐1)‐induced hypophysitis was less common than that induced by combination therapy with PD‐1 and cytotoxic T‐cell antigen‐4 inhibitors [22]. Considering the extremely low frequency of hypophysitis (secondary adrenocortical insufficiency: 0.3%) in CheckMate 274 [2], substantial discrepancies exist between the RCT and our real‐world data. One possible explanation is that perhaps the Asian population may have an increased risk of hypophysitis occurrence. In a prospective study assessing 174 Japanese patients with malignant melanoma and lung cancer, 6% treated with PD‐1 developed pituitary‐irAE, with all exhibiting isolated ACTH deficiency [23]. The authors highlighted the possibility of pituitary‐irAE being overlooked, since symptoms associated with pituitary‐irAE such as fatigue, appetite loss, and weight loss were nonspecific and similar to those with malignancy. In our study, at least three patients presented with isolated ACTH deficiency. Another potential explanation for this disparity in these endocrine AEs includes that patients in the adjuvant setting may be more immunocompetent, possibly increasing the risk of irAEs. In support of this, the proportion of patients who discontinued treatment due to irAEs was higher in the adjuvant setting (16%) than in the metastatic setting (6%) [8, 24]. Despite the mechanism remaining to be elucidated, hypophysitis induced by anti‐PD‐1 therapy is characterized by isolated and severe ACTH deficiency with the time to onset ranging widely from a few weeks to more than a year later [25]. In fact, the time to ≥G3 of these irAEs ranged variably from 3.3 to 18.3 months. Since these irAEs require prolonged hormone replacement therapy, which causes a significant burden and impairs quality of life, clinicians should keep them in mind when patients have or had received anti‐PD‐1 therapy [26].

Our study is not devoid of limitations. First, the lack of a standardized protocol, including surgical techniques, evaluation of irAE, and decisions to NAC use and regimens, etc., and central pathological review inherent to the retrospective nature may have introduced potential bias. Second, the limited number of patients in this study may have been underpowered for detection of differences between the BC and UTUC groups, or the NAC and non‐NAC groups, suggesting the need for further large‐scale studies to validate our findings. In addition, since this was a retrospective multicenter study without a unified data cutoff date, follow‐up data varied across institutions. This heterogeneity in follow‐up duration may have influenced the outcome. Third, our study failed to collect data on patients who opted for observation or adjuvant chemotherapy use among eligible patients (pT3‐pT4a and/or pN+, or ypT2‐ypT4 and/or ypN+) during the study period. As such, our study could not fully capture patients with MIUC who need additional treatment after surgery. Fourth, patients with UTUC who did not perform lymph node dissection may harbor occult nodal metastases, which may introduce potential bias due to stage migration. Fifth, given that a previous study using a different cut‐off value (100/μL) [16], different cut‐off values may have resulted in different outcomes, necessitating the external validation in further prospective studies to ensure the prognostic role and optimal cut‐off of AEC.

In conclusion, we found that adjuvant nivolumab therapy provided comparable oncological outcomes between patients with BC and UTUC. Given the considerable percentage of patients with severe renal impairment following extirpative surgery, adjuvant nivolumab therapy may be a practical option for patients with UTUC at high‐risk of recurrence. Also, an elevated AEC was an independent prognostic factor for worse DFS. In addition, we found a higher incidence of endocrine irAEs, such as adrenal insufficiency and hypophysitis, with a variable onset time. Clinicians should take the possibility of these irAEs into consideration when patients who have received or have a history of anti‐PD‐1 therapy suffer from nonspecific symptoms such as fatigue or appetite loss.

## Author Contributions

**Satoshi Katayama:** conceptualization, methodology, data curation, formal analysis, writing – original draft. **Tatsushi Kawada:** methodology, conceptualization. **Keiichiro Mori:** conceptualization, methodology. **Kosuke Iwatani:** data curation, investigation. **Shota Inoue:** data curation, investigation. **Tomofumi Watanabe:** investigation, data curation. **Ichiro Tsuboi:** investigation, data curation. **Tomoaki Yamanoi:** data curation, investigation. **Takehiro Iwata:** validation, investigation. **Kazuma Tsuboi:** data curation, investigation. **Wataru Murao:** data curation. **Tetsuya Nakada:** data curation. **Kohei Ogawa:** data curation. **Akihiro Matsukawa:** data curation. **Takafumi Yanagisawa:** validation, writing – review and editing. **Shingo Nishimura:** data curation, investigation, project administration. **Kensuke Bekku:** validation. **Koichiro Wada:** supervision. **Takahiro Kimura:** supervision. **Motoo Araki:** supervision, writing – review and editing.

## Ethics Statement

This study was approved by the ethics committee of Okayama University hospital (K2505‐009) and subsequently approved each institutional review board at all participating institutions.

## Consent

An opt‐out methodology was implemented, in which patients were provided the opportunity to decline participation.

## Conflicts of Interest

Koichiro Wada, Takahiro Kimura, and Motoo Araki are members of the Editorial Board of *International Journal of Urology* and the co‐authors of this article. To minimize bias, they were excluded from all editorial decision‐making related to the acceptance of this article for publication. The other authors declare no conflicts of interest.

## Supporting information


**Supplementary Figure 1.** Patients' flow diagram.
**Supplementary Figure 2**. Kaplan–Meier analysis of disease‐free survival stratified by NAC exposure in the whole (A), bladder carcinoma (B), and upper tract urothelial carcinoma groups (C).
**Supplementary Figure 3**. Kaplan–Meier analysis of disease‐free survival among patients with high‐risk muscle invasive urothelial carcinoma treated with adjuvant nivolumab therapy harboring both T and N stages, T stage only, or N stage only.

**Supplementary Table 1.** Details of subsequent therapy for disease recurrence in patients with muscle‐invasive urothelial carcinoma receiving adjuvant nivolumab therapy.
**Supplementary Table 2**. Multivariable analyses assessing factors associated with disease‐free survival in a subgroup of patients excluding those without lymph node dissection (pNx).

## Data Availability

The data that support the findings of this study are available from the corresponding author upon reasonable request.
