# Supplementary material for: Oncological Outcomes and Safety Profiles of Adjuvant Nivolumab in Muscle‐Invasive Urothelial Carcinoma
Source: Int J Urol. 2026 Oct 19;33(10):e70664. doi: 10.1111/iju.70664 (PMC13680791; doi:10.1111/iju.70664)
Supplement: Supplementary file 1 [file IJU-33-0-s001.docx]

| **Supplementary Table 1.**  Details of subsequent therapy for disease recurrence in patients with muscle-invasive urothelial carcinoma receiving adjuvant nivolumab therapy | |
| --- | --- |
| **Number of overall patients** | 97 (100%) |
| **Number of patients who experienced recurrence** | 50 (52%) |
| **Number of patients treated with subsequent therapy** | 41 (42%) |
| Enfortumab vedotin | 17 (18%) |
| Platinum-based chemotherapy | 14 (14%) |
| Pembrolizumab | 5 (5%) |
| Others | 5 (5%) |

**Supplementary Table 2.** Multivariable analyses assessing factors associated with disease-free survival in a subgroup of patients excluding those without lymph node dissection (pNx).

|  | **DFS** | | | | |
| --- | --- | --- | --- | --- | --- |
|  |  | Multivariable | | | |
|  |  | HR | (95% CI) | *P* | |
| **Neoadjuvant chemotherapy** |  | 0.86 | 0.36-2.06 | 0.74 | |
| **Pathological T stage** (ref. ≤ ypT1/pT2) |  | 0.80 | 0.23-2.84 | 0.73 | |
| **Absolute eosinophil count**  (ref. < 239.7) |  | 1.95 | 0.94-4.07 | 0.07 | |
| **UTUC** (ref. bladder) |  | 2.38 | 0.87-6.57 | 0.09 | |
| Bold P values are considered statistically significant. *DFS* disease-free survival. *UTUC* upper tract urothelial carcinoma. | | | | |  |
