# Supplementary material for: Oncological Outcomes and Safety Profiles of Adjuvant Nivolumab in Muscle‐Invasive Urothelial Carcinoma
Source: Int J Urol. 2026 Oct 19;33(10):e70664. doi: 10.1111/iju.70664 (PMC13680791; doi:10.1111/iju.70664)
Supplement: Supplementary file 2 [file IJU-33-0-s002.docx]

**Supplementary figure 1.** Patients’ flow diagram

**Supplementary figure 2.** Kaplan-Meier analysis of disease-free survival stratified by NAC exposure in the whole (A), bladder cancer (B) and upper tract urothelial carcinoma groups (C)

*P* =0.92

(C)

*P* =0.54

*P* =0.34

(A)

(B)

**Supplementary figure 3.** Kaplan-Meier analysis of disease-free survival among patients with high-risk muscle invasive urothelial carcinoma treated with adjuvant nivolumab therapy harboring both T and N stages, T stage only, or N stage only.
